# Oxygen saturation targets in neonatal care: A narrative review

**DOI:** 10.1016/j.earlhumdev.2024.106134

**Published:** 2024-10-28

**Authors:** Tri C. Nguyen, Rajeshwari Madappa, Heather M. Siefkes, Michelle J. Lim, Kanya Mysore Siddegowda, Satyan Lakshminrusimha

**Affiliations:** a Kaiser Permanente North California, 1640, Eureka Rd, Roseville, CA 95661, USA; b Department of Pediatrics, SIGMA Hospital, P8/D, Kamakshi Hospital Road, Mysore 570009, India; c Department of Pediatrics, UC Davis Children’s Hospital, 2516 Stockton Blvd, Sacramento, CA 95817, USA

**Keywords:** Oxygen, Free radicals, Pulmonary vascular resistance, Hypoxia, Hyperoxia

## Abstract

Optimal oxygenation requires the delivery of oxygen to meet tissue metabolic demands while minimizing hypoxic pulmonary vasoconstriction and oxygen toxicity. Oxygen saturation by pulse oximetry (SpO_2_) is a continuous, non-invasive method for monitoring oxygenation. The optimal SpO_2_ target varies during pregnancy and neonatal period. Maternal SpO_2_ should ideally be ≥95 % to ensure adequate fetal oxygenation. Term neonates can be resuscitated with an initial oxygen concentration of 21 %, while moderately preterm infants require 21–30 %. Extremely preterm infants may need higher FiO_2_, followed by titration to desired SpO_2_ targets. During the NICU course, extremely preterm infants managed with an 85–89 % SpO_2_ target compared to 90–94 % are associated with a reduced incidence of severe retinopathy of prematurity (ROP) requiring treatment, but with higher mortality. During the later stages of ROP progression, studies suggest that higher SpO_2_ targets may help limit progression. A target SpO_2_ of 90–95 % is generally reasonable for term infants with respiratory disease or pulmonary hypertension, with few exceptions such as severe acidosis, therapeutic hypothermia, and possibly dark skin pigmentation, where 93–98 % may be preferred. Infants with cyanotic heart disease and single-ventricle physiology have lower SpO_2_ targets to avoid pulmonary over-circulation. In low- and middle-income countries (LMICs), the scarcity of oxygen blenders and continuous monitoring may pose a challenge, increasing the risks of both hypoxia and hyperoxia, which can lead to mortality and ROP, respectively. Strategies to mitigate hyperoxia among preterm infants in LMICs are urgently needed to reduce the incidence of ROP.

## 1. Introduction

Oxygen is the elixir of life and optimal delivery of oxygen is the essence of neonatal critical care. Oxygen is one of the most commonly used drugs in the Neonatal Intensive Care unit (NICU). Oxygen therapy in neonatal care has four goals. Delivery of oxygen to the tissues to avoid anerobic metabolism and lactic acidosis, enabling growth and development, minimizing hypoxic pulmonary vasoconstriction and oxygen toxicity. [1] The targets for oxygen saturation change with age and pathophysiological state and have been studied extensively in the neonatal, pediatric and adult critical care units from preterm infants to adults with acute respiratory distress syndrome (ARDS). [2] This review focuses on the physiology of oxygen delivery, basics of pulse oximetry, alarms and targets for oxygen saturation (SpO_2_), arterial oxygen pressure (PaO_2_) and fraction of inspired oxygen (FiO_2_) in various maternal and neonatal disease states. We review the history of oxygen use in the NICU and summarize clinical studies evaluating optimal targets in preterm. Finally, we end with a discussion on challenges to maintain oxygen targets in resource limited settings in low-and-middle income countries (LMIC).

## 2. Physiology of oxygen delivery

Oxygen transport is fundamental to aerobic metabolism and essential for cellular functions of all complex living organisms. Oxygen delivery is the rate at which oxygen is transported from the lungs to peripheral tissues for cellular uptake. The lungs, heart, vasculature and red blood cells all play a pivotal role in delivery and transport of oxygen to peripheral tissues. Venous blood normally returns to the lungs deoxygenated, at a partial pressure of ~40 mmHg (~ 5.3 kPa). Blood is passed through the pulmonary capillaries where oxygen and carbon dioxide are equilibrated across the alveolar-capillary-barrier, resulting in removal of carbon dioxide and oxygen uptake. Oxygenated blood leaving the lungs and returning to the heart has a partial pressure of ~100 mmHg (~ 13.3 kPa) of oxygen. Hemoglobin (Hb) is the primary carrier of oxygen molecules to peripheral tissues, with 98 % of total oxygen transported bound to hemoglobin and 2 % dissolved in plasma.

Oxygen delivery to the tissues $\left({DO}_{2}\right)$ is dependent on oxygen content of arterial blood $\left({CaO}_{2}\right)$ and blood flow to the tissues. Arterial oxygen content is based on hemoglobin level and oxygen saturation $\left({CaO}_{2}\right)$. Oxygen delivery can be estimated with the following equation: ${DO}_{2}\;(mL/min)=Q\times {CaO}_{2}$; Q=cardiacoutput and ${CaO}_{2}=\text{concentration}\;\text{of}\;\text{oxygen}\;\text{in}\;\text{arterial}\;\text{blood}$.

${CaO}_{2}=1.34^{*}{Hb}^{*}\left({SaO}_{2}/100\right)+0.003^{*}{PaO}_{2}$, where Hb=hemoglobiningramsperdeciliter, ${SaO}_{2}=\text{percentage}\;\text{of}\;\text{saturated}\;\text{hemoglobin}\;\text{with}\;\text{oxygen}$, ${PaO}_{2}=\text{partial}\;\text{pressure}\;\text{of}\;\text{oxygen}\;\text{in}\;\text{the}\;\text{blood}\;\text{in}\;\text{mmHg}$. The oxygen carrying capacity of hemoglobin is estimated to be 1.34 mL O_2_/g Hb and the solubility coefficient of oxygen is estimated to be 0.003.

Oxygen delivery can be impacted by low cardiac output, anemia, lung disease, intracardiac shunt, low inspired oxygen or hypoventilation. In the setting of diminished oxygen delivery, normal oxygen consumption (VO_2_) can be maintained by a compensatory increase in oxygen extraction from the periphery. However, critical DO_2_ is reached when oxygen extraction exceeds its critical threshold and can no longer compensate for reduced oxygen delivery, resulting in tissue hypoxia, anaerobic metabolism and lactic acidosis. At these pathological states, VO_2_ is dependent on DO_2_. Maintaining DO_2_ above this critical point is an essential component of intensive care unit management. [2] Below the critical point, anerobic metabolism occurs resulting in increased lactic acid and decreasing venous oxygen saturation.

**Fig. 1.**
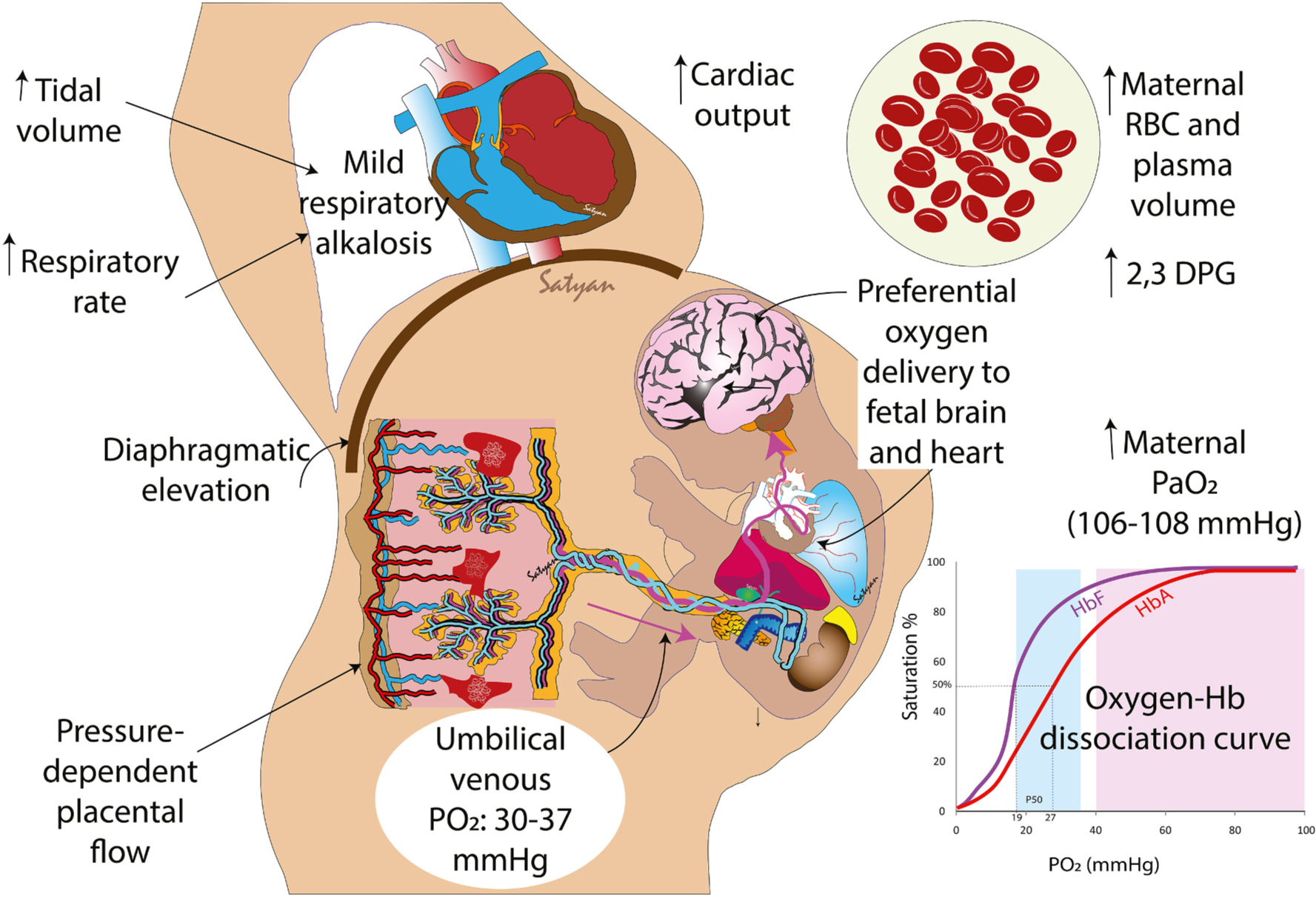
Maternal and fetal oxygen transport. Changes during pregnancy, such as increased tidal volume, higher respiratory rate and elevated diaphragm result in mild respiratory alkalosis. Increased cardiac output, higher RBC and plasma volume and elevated 2,3 diphosphoglycerate (2,3-DPG) contribute to higher oxygen content and delivery to the fetus. These factors result in higher maternal PaO_2_ compared to non-pregnant women. Due to relative differences in the pattern of the oxygen-hemoglobin dissociation curves (inset), the fetus is able to extract adequate oxygen and preferentially deliver it to fetal tissues, especially the brain, heart and other vital organs.

Factors influencing arterial blood flow include blood pressure, cardiac contractility and peripheral vascular resistance. In addition, cerebral blood flow is inversely related to PaCO_2_. [3] Both the quantity and type of hemoglobin influence oxygen carrying capacity and delivery to tissues. Having a higher hemoglobin increases CaO_2_ and DO_2_. However, studies evaluating a higher vs. a lower threshold for transfusion (with HbA containing packed RBCs) and maintaining a higher hemoglobin have not led to any difference in survival without neurodevelopmental impairment in preterm infants. [4] However, delayed cord clamping (DCC) is associated with higher fetal hemoglobin levels and lower mortality compared to early cord clamping. [5] It is not clear if these differences are due to the type of hemoglobin (adult HbA vs. fetal HbF) or source (stored adult blood vs. fresh fetoplacental blood). The oxygen dissociation curve of HbA is shifted to the right compared to HbF (Fig. 1 inset) and hence has less affinity for oxygen and is able to effectively deliver oxygen to tissues. However, HbF is more effective in oxygen delivery during periods of hypoxemia (similar to fetal state as is typical in infants with severe persistent pulmonary hypertension of the newborn-PPHN) [6].

## 3. Pulse oximeter basics

The gold standard measure of oxygenation is an arterial blood gas with partial pressure of oxygen (PaO_2_) and corresponding saturation by co-oximetry (SaO_2_). SaO_2_ is the percentage of oxyhemoglobin concentration relative to total hemoglobin concentration in the arterial blood and it is an important clinical parameter. However, measuring PaO_2_/SaO_2_ is invasive and not always feasible. Pulse oximetry provides a noninvasive measurement of peripheral arterial oxygen saturation (SpO_2_) and can be measured continuously. Pulse oximetry uses photoplethysmography (PPG) in red and infrared wavelengths, which oscillate as peripheral arterial volume changes (or pulses) throughout the cardiac cycle. Due to the differences in light absorption of oxyhemoglobin (HbO_2_) and deoxyhemoglobin (deHb) at these wavelengths, the oximeters can estimate arterial oxygen saturation. [7].

In addition to SpO_2_, oximeters can provide clinically important parameters continuously such as heart rate, which can be beneficial during resuscitation in the delivery room. Oximeters can also provide parameters related to perfusion, such as perfusion index, due to the use of PPG that pulses as arterial blood volume changes. Perfusion index is essentially the amplitude of the PPG waveform and reflective arterial volume, however routine clinical use of perfusion related parameters from oximeters are still early in investigation. [7,8].

**Fig. 2.**
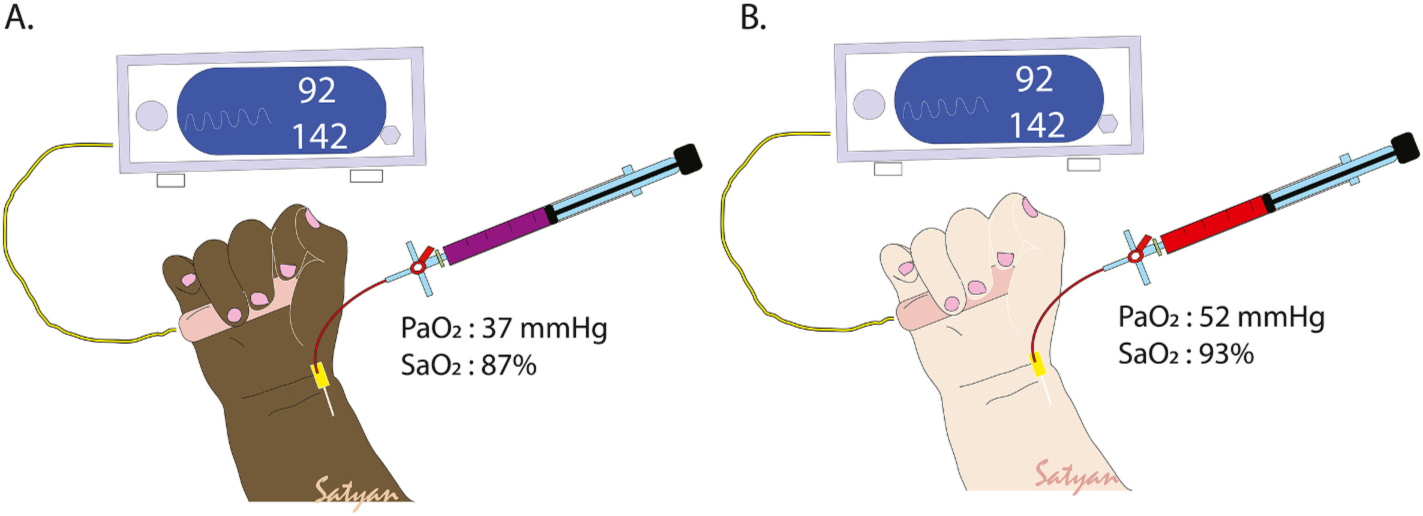
Occult hypoxemia and skin pigmentation. Occult or hidden hypoxemia refers to situations where oxygen saturation by pulse oximeter is ≥90 % but oxygen saturation by co-oximetry on an arterial blood gas is <90 % (or <88 % in some references). Occult hypoxemia is more common in subjects with darker skin pigmentation (A) compared to less pigmentation (B).

Clinicians should be aware that the accuracy of SpO_2_ relative to SaO_2_ decreases with hypoxemia, and oximeters may then underestimate the degree of hypoxemia. There are also recent concerns regarding accuracy so SpO_2_ relative to SaO_2_ in darker pigmented patients resulting in “hidden” or occult hypoxemia (Fig. 2), however the impact of this in neonates is unknown currently and is under investigation (NCT 06063148, PI-Siefkes) [9].

## 4. Maternal oxygenation and fetal wellbeing

The maintenance of adequate fetal oxygenation to preserve fetal viability and sustain fetal growth throughout pregnancy involves a complex interrelationship between mother, placenta, and the developing fetus. [10,11] The unique characteristics of the placenta as well as critical maternal and fetal adaptations allow for enhancement of fetal oxygenation.

Maternal adaptations during pregnancy include respiratory changes that assist in fetal oxygenation (Fig. 1). During the first trimester of pregnancy, increased progesterone cause both an increase in maternal tidal volume and respiratory rate by 30–50 %. These changes result in an increase in maternal PaO_2_ to 106–108mmHg (~14.1 kPa) and a decrease in maternal PaCO_2_ to ~30 mmHg (~ 4 kPa). This results in slight respiratory alkalosis with associated metabolic renal compensation. There is also an increase in maternal red blood cell production of 2,3-diphosphoglycerate (2,3-DPG) leading to a rightward shift of the maternal oxygen dissociation curve promoting maternal oxygen delivery to the fetus.

By comparison, the developing fetus has a relative hypoxic environment, with normal fetal umbilical venous PO_2_ to be 30 to 37mmHg (4 to 5 kPa). Adequate oxygen delivery to the developing fetus is determined both by maternal oxygen carrying capacity and uterine blood flow to the placenta. Placental blood flow is not hormonally autoregulated and entirely pressure dependent. Oxygenated maternal blood goes through the placenta via uterine arteries into the intervillous space of the placenta. Oxygen then crosses the placental membrane and enters the umbilical vein, supplying oxygen to the fetus. Oxygen molecules move down a pressure gradient via simple diffusion from the maternal arterial side to the fetal venous side. The low partial pressure of fetal venous oxygen coupled with the high oxygen carrying capacity and affinity of fetal blood allows for adequate oxygenation for growth and development. Further, high fetal cardiac output, high fetal hemoglobin concentration, and presence of circulatory shunts (foramen ovale, ductus arteriosus, ductus venosus) allow for a unique preferential distribution of oxygenated blood to the heart, brain, and other vital organs (Fig. 1). [12].

Acute reductions on uteroplacental blood flow or maternal oxygen delivery (maternal hypoxia or hypotension) will result in fetal hypoxia and distress. Long-term sequelae of fetal hypoxia may result in fetal or intrauterine growth restriction (FGR or IUGR) and compromise in neurodevelopment. For this reason, maternal oxygen supplementation [13,14] has been utilized routinely in clinical practice for suspected fetal distress and prophylactically during the second stage of labor as well as proposed in clinical scenarios of IUGR and congenital heart disease. However, empiric evidence to support routine use of oxygen support in the setting of fetal distress is lacking and remains somewhat controversial in practice. [14] Early studies have shown administration of sub-atmospheric oxygen to pregnant women compared to 21 % oxygen support, resulted in depressed fetal activity. [15] However, more recent clinical trials looking at empiric oxygen administration during labor, demonstrated no difference in abnormal cord blood pH (< 7.2) in women treated with oxygen support vs control [14], although previous studies suggested increased fetal acidosis with maternal oxygen supplementation. [16] Maternal oxygen supplementation can lead to a drop in partial pressure of carbon dioxide. [17] Concerns surrounding oxygen support, especially in setting of normal maternal oxygen levels, are related to its rather unknown effects on placental gas exchange, fetal acid-base equilibrium and possible vasoconstrictive effects on umbilical and placental vessels. Generation of radical oxygen species and oxygen toxicity to developing fetal cells is also of theoretical concern.

## 5. Neonatal resuscitation –late-preterm/term, moderately preterm and extremely preterm gestational ages

**Fig. 3.**
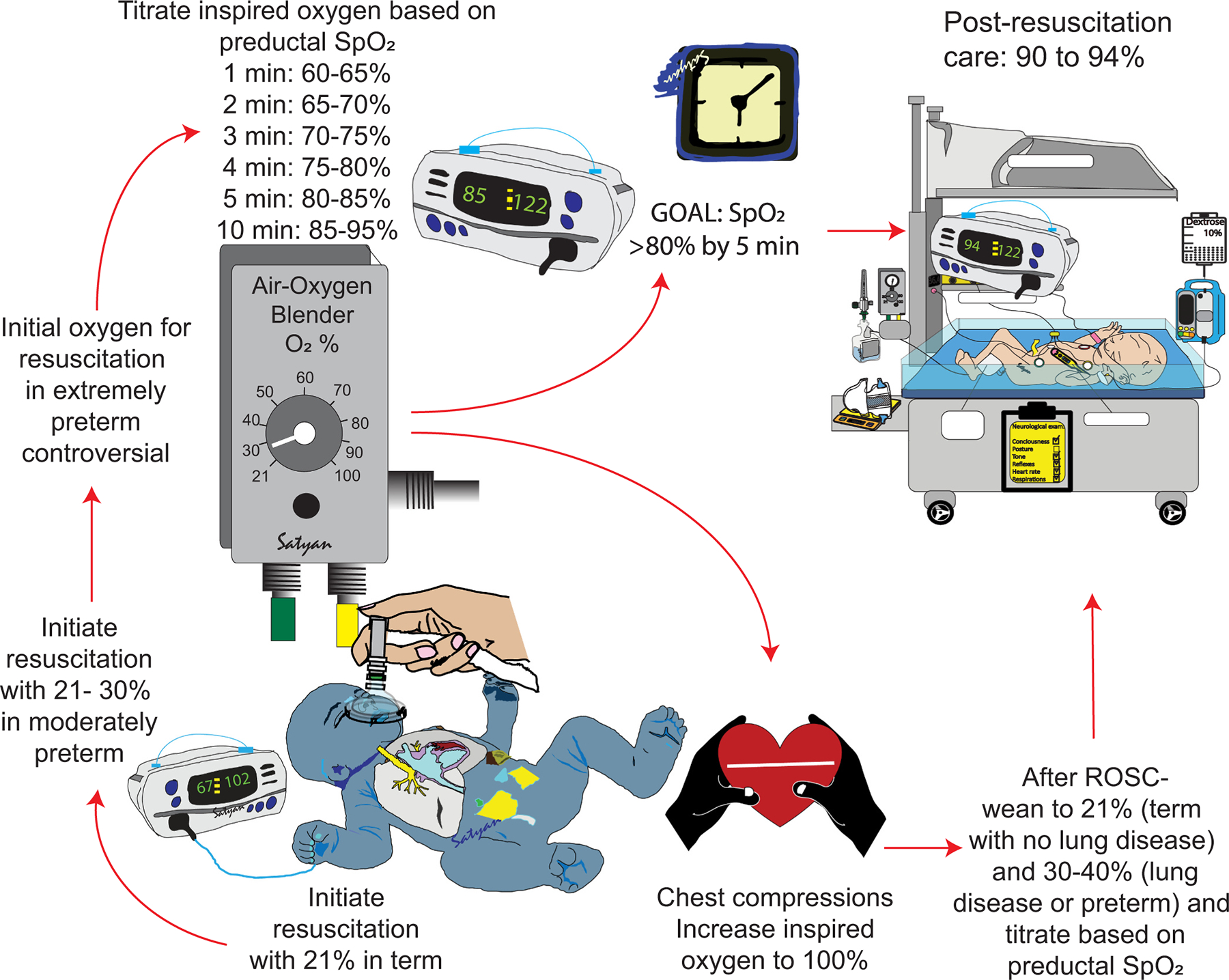
Initial inspired oxygen concentration and oxygen saturation targets during neonatal resuscitation. The optimal initial concentration of oxygen is 21 % for term infants and 21–30 % for preterm infants. However, among extremely preterm infants, the initial concentration of oxygen is not clear as recent evidence suggests lower mortality with high oxygen. During resuscitation, it is recommended to target goal SpO_2_ as recommended by the neonatal resuscitation program. [95,96] During chest compressions, it is recommended that inspired oxygen be increased to 100 %. Following return of spontaneous circulation (ROSC), abrupt weaning to 21 % (term, no lung disease) or 30–40 % (preterm or with lung disease) may reduce oxygen exposure and toxicity. The goal of resuscitation is to achieve a preductal SpO_2_ of at least 80 % by 5 min as failure to achieve it results in increased mortality and morbidity. Based on [98].

The optimal oxygen concentration for resuscitation of late-preterm and term infants >35 weeks of gestation is 21 % oxygen (Fig. 3). [18,19] Systematic reviews have demonstrated higher mortality with the use of 100 % oxygen for initial resuscitation at this gestational age. [20] Animal models have shown excessive arterial contractility and superoxide anion formation in pulmonary arteries and subsequent impairment of response to inhaled nitric oxide (iNO) following resuscitation with 100 % oxygen. [21–23] Initiating resuscitation with 21 % oxygen and titrating to achieve preductal SpO_2_ goals set up the neonatal resuscitation program (NRP) in asphyxiated lambs with meconium aspiration results in optimal pulmonary blood flow similar to that achieved in control term lambs. [24].

For preterm infants <32 weeks gestational age, the optimal initial concentration of oxygen for resuscitation is not known. [25] A recent individual participant data network meta-analysis suggested that with low certainty of evidence, initial resuscitation with 100 % oxygen was associated with lower mortality than intermediate (50–65 %) or low (21–30 %) oxygen. [26] While it is too early to make strong recommendations, especially for extremely low birth weight infants <28 weeks gestation, [27] there is considerable uncertainty regarding the continued use of 21–30 % oxygen for initial resuscitation of these extremely premature infants. [28] The focus of resuscitation at any gestational age group is to achieve preductal SpO_2_ of >80 % by 5 min after birth. [29,30] Further studies on oxygen titration are needed to figure out rapidity of FiO_2_ adjustment during resuscitation.

It is recommended that infants requiring chest compressions received 100 % oxygen during positive pressure ventilation (PPV). [18] During chest compressions for bradycardia or asystole, the pulmonary, coronary and cerebral blood flow are very low and increasing the oxygen content by providing 100 % oxygen might enhance oxygen delivery to these organs. [31] Once return of spontaneous circulation is achieved, oxygen should be weaned to achieve target SpO_2_. Studies in asphyxiated lamb models have suggested that abrupt weaning to 21–30 % oxygen and then titrating FiO_2_ based on SpO_2_ after ROSC minimizes hyperoxia and oxidative stress markers compared to slow wean from 100 %.[32,33].

## 6. Post-resuscitation phase

There are few clinical studies on optimal oxygenation during the post-resuscitation period. Kapadia et al. have demonstrated that high PaO_2_ (>100–114 mmHg or > 13.3 to 15.2 kPa) on the first arterial gas after admission for perinatal distress is associated with higher incidence of hypoxic ischemic encephalopathy (HIE) compared to PaO_2_ < 100 mmHg (13.3 kPa). [34] Weaning FiO_2_ and minimizing the risks of hyperoxia are important for optimal neurological outcome following resuscitation for perinatal asphyxia. However, perinatal asphyxia is commonly associated with PPHN, and hypoxia (PaO_2_ < 45–50 mmHg or 6 to 6.7 kPa) increases pulmonary vascular resistance (PVR). [35] This leaves neonatal providers with a narrow therapeutic window for preductal PaO_2_ (50–100 mmHg or 6.7 to 13.3 kPa) or approximately 90–97 % preductal SpO_2_ during the post-resuscitation phase. [35].

## 7. Oxygen saturation targets in the NICU

### 7.1. Preterm infants

The history of oxygen use in preterm infants and associated retinopathy of prematurity (ROP) offers important lessons and will be covered in this section followed by a discussion of optimal SpO_2_ targets in this population.

#### 7.1.1. Historical perspective: oxygen and retinopathy of prematurity

The optimal range for SpO_2_ in preterm infants remains unknown despite its use in newborn resuscitation as early as 1928. It was not until 1934 that Dr. Julius Hess, Chief of Pediatrics at the Micheal Reese Hospital in Chicago created the very first inhaled oxygen delivery device for infants and young children. [36] This heralded in decades of routine and unrestricted use of oxygen in the newborn period [37]. In 1942, the very first publication by Terry documented numerous cases of a progressive disorder of the eye seen exclusively in the premature infants. [38]. In fully developed cases, a vascularized grayish membrane was observed behind the eye. He coined these findings retrolental fibroplasia (RLF), which is now recognized as advanced stage of ROP. It was believed that the retrolental membrane developed from the abnormal growth of the fetal embryonic vitreous [39,40]. It wasn’t until the mid-1940s that Drs. Owens (husband and wife ophthalmologist team) at Johns Hopkins Hospital and University started to routinely examine and follow up weekly on all premature infants with birth weights of 2000 g or systematically evaluate ROP [41]. In the mid-1950s, work by Kinsey and Campbell showed that oxygen was the main culprit behind the crippling eye disease that contributed to the leading cause of blindness in children of preschool age at that time [42–44]. To this day, these findings have contributed to the swinging pendulum of oxygen use as clinicians try to increase survival in the most premature infants while balancing the risk of ROP.

#### 7.1.2. Clinical trials evaluating supplemental oxygen in preterm infants

To this day, the optimal supplemental oxygen and target SpO_2_ during resuscitation and NICU course of preterm infants remains controversial [45]. After the discovery of ROP, several small clinical studies in the 1950s recommended restricted use of supplemental oxygen [46,47]. Those studies showed a trend towards increased mortality in the oxygen-restricted infants but because the findings did not show statistical significance, restricted oxygen use continued. These studies suggested estimates of 16 additional deaths for every case of blindness prevented [48]. In a separate meta-analysis of 5 early trials (1951–1969) on the effects of lower versus higher oxygen concentration in preterm infants, Askie and Henderson-Smart found that restriction of oxygen reduced the incidence and severity of ROP without increasing mortality. Furthermore, they calculated that to prevent one infant from having an adverse outcome of death or RLF, 3 infants needed restricted oxygen therapy [49]. Unfortunately, all these studies were undertaken in the era without continuous pulse oximetry.

In the 1990s and 2000s, several randomized controlled trials (RCT) aimed to look at the effect of differential oxygen target saturations. The STOP-ROP (Supplemental Therapeutic Oxygen for Prethreshold Retinopathy of Prematurity) study randomized 649 infants from 30 centers over a 5-year period to either a SpO_2_ range of 89 % to 94 % (conventional arm) or 96 % to 99 % (supplemental arm) when they reached prethreshold ROP (using International Classification of Retinopathy of Prematurity-ICROP classification of 1984). Although progression to threshold ROP was not significantly different between the two groups, a subgroup analysis demonstrated significant benefits for infants in the 96–99 % oxygen saturation arm who did not have “plus disease” (defined as at least 2 quadrants of dilation and tortuosity of the posterior pole vessels). However, those infants in the liberal oxygen arm experienced an increased risk of adverse pulmonary events such as pneumonia, exacerbation of chronic lung disease and the need for oxygen, diuretics and hospitalization at 3 months of corrected age [50,51].

In 2003, a second RCT, Benefits of Oxygen Saturation Targeting (BOOST), hypothesized that maintaining higher oxygen saturation target ranges would improve overall growth and neurodevelopmental outcomes at 12 months corrected age. This study assigned all eligible infants born <30 weeks’ gestation who were still on supplemental oxygen at 32 weeks’ gestation to a target functional oxygen saturation range of either 91 to 94 % (standard group) or 95 to 98 % (high-saturation group). The study reported no significant benefits in the higher saturation range, but similar to the STOP-ROP trial, infants in the higher oxygen saturation range had increased length of oxygen requirement and oxygen dependency at 36 weeks corrected, along with a higher frequency of home oxygen need [52].

To address the uncertainty of optimal oxygen saturation target in extremely preterm infants (<28 weeks’ postmenstrual age), an international team of experts gather in 2003 to form the Neonatal Oxygenation Prospective Meta-Analysis (NeOProM) collaborative. It was a prospective study that incorporated 5 separate clinical trials: SUPPORT (Surfactant, Positive Pressure and Pulse Oximetry Randomized Trial) in the United States, the 3 BOOST-II (Benefits of Oxygen Saturation Target) trials from the United Kingdom, Australia and New Zealand, and the COT (Canadian Oxygen Trial). Although each trial had slight variations, all studies examined the effect of lower-target oxygen saturation (85 % to 89 %) and higher-target oxygen saturation (91 % to 95 %) in infants born before 28 weeks’ gestation, with the primary outcome of composite death or disability at 18 to 24 months of corrected age. Such a large prospective study (4751 patients) was required to have an 80 % power to detect a 4 % difference in the rate of death or disability [53].

The SUPPORT trial (*n* = 1316) randomized infants between 24 ^0/7^ weeks’ and 27^6/7^ weeks’ gestational age to the 2 different oxygen saturation arms and to either CPAP or intubation and surfactant within 2 h of age. The primary outcome was a composite of severe ROP, death before discharge from the hospital, or both. The composite primary outcome did not differ significantly between the lower and the higher oxygen saturation target groups however death before discharge from the NICU was significantly higher (19.9 %) in the lower oxygen saturation group when compared to the higher oxygen saturation group (16.2 %) with a number needed to harm of 27 (RR, 1.27 [95 % CI, 1.01–1.60; *P* = 0.04]). At 18 to 22 months of corrected age, mortality was still significantly higher in the lower oxygen saturation group (22.1 % vs 18.2 %; RR 1.25 [95 % CI, 1.00–1.25]). However, the rate of severe ROP (defined as presence of threshold retinopathy, need for surgical intervention or use of bevacizumab) in survivors was 8.6 % in the lower oxygen saturation group compared to 17.9 % in the higher oxygen saturation group (RR, 0.52 [95 % CI, 0.37–0.73; *P* < 0.001]) [54].

The BOOST-II trials, which included the United Kingdom, Australia and New Zealand randomized preterm infants in the first 24 postnatal hours (later than the SUPPORT trial). A total of 2448 infants were enrolled in the 3 clinical trials from March 2006 to December 2010. In early 2009, it was discovered by investigators in the United Kingdom that during the trials, the standard Masimo Radical oximeters that were being used in all 5 trials, had an artifact in the algorithm causing SpO_2_ values between 87 % to 90 % to read 1–2 % higher, thus leading to less frequent readings of 87 % to 90 %. [55] Therefore, halfway through the trials, a total of 1187 infants changed over to the revised oximeter-calibration algorithm. Of the 1187 infants whose treatment used the new algorithm, the rate of death was significantly higher in the lower-oxygen target saturation group (23.1 % vs. 15.9 %); (RR, 1.45 [95 % CI, 1.15 to 1.84; *P* = 0.002]). These findings suggested that 14 infants to be treated with higher oxygen-saturation target to prevent 1 death. However, in all the overall combined data (2448 infants), there was no significant difference in rate of death in the lower-target oxygen group as compared to the higher-target oxygen group (19.2 % vs. 16.6 %; (RR 1.16, [95 % CI, 0.98 to 1.37; *P* = 0.09) but those in the lower-oxygen saturation target group had a reduced rate of ROP (10.6 % vs. 13.5 %; (RR 0.79; [95 % CI, 0.63 to 1.00; *P* = 0.045]) and an increase rate of necrotizing enterocolitis (10.4 % vs. 8.0 %); (RR, 1.31 [95 % CI, 1.02 to 1.68; P = 0.04]) [56]. In 2016, long term follow-up results from the BOOST-II Australia and UK collaborative group concluded that the use of oxygen saturation target range of 85 to 89 % vs. 91 to 95 % resulted in no significantly higher rates of death or disability at 2 years of age in each trial but when combined in a post hoc analysis, there was significant increase in the combined outcome of death and disability and of death alone [57]. When data combined from all 3 BOOST-II sites were analyzed by Cummings et al., no significant difference in this outcome between the lower [46.8 % (557 death out of 1189)] vs. higher oxygen saturation group [43.4 % (513 death out of 1181)]; *P* = 0.10) were observed [58]. Lastly, in the COT trial (*N* = 1147), the rates of death (16.6 % in the lower vs. 15.3 % in the higher target oxygen group (OR 1.11; [95 % CI, 0.80 to 1.54); *P* = 0.54)] and death or disability at 18 months of age were similar [59]. In a prospectively planned meta-analysis of individual participant data from all 5 trials, Askie et al. evaluated 4965 infants concluded that there was no difference in the primary outcome of death or major disability at a corrected age of 18–24 months. However, the lower SpO_2_ target range was associated with higher risk of death and necrotizing enterocolitis (NEC), but a lower risk of ROP treatment. [60].

#### 7.1.3. Oxygen and ROP: a biphasic disease requiring changing SpO_2_ targets

**Fig. 4.**
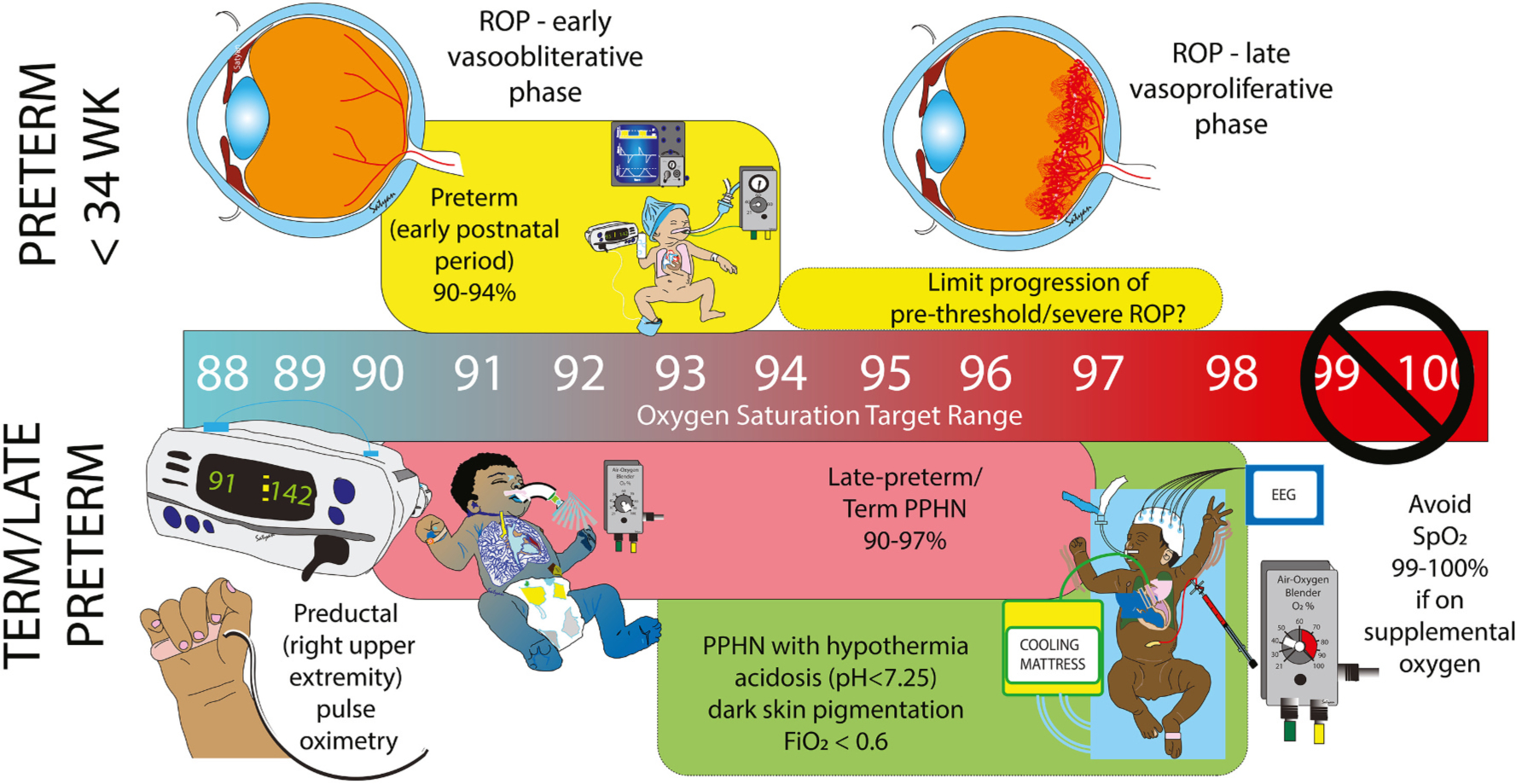
Recommended preductal oxygen saturation in the NICU. Preductal oxygen saturation by pulse oximetry (SpO_2_) is the preferred mode of non-invasive and continuous assessment of oxygenation in the NICU. We recommend a target of 90–97 % in term infants and late preterm infants with persistent pulmonary hypertension of the newborn (PPHN). Ranges recommended in preterm infants without pulmonary hypertension (PH), with PH and bronchopulmonary dysplasia (BPD), and those with progression of retinopathy of prematurity (ROP) are shown. In term infants with PPHN, hypothermia, acidosis, dark skin pigmentation and low FiO_2_ need, should consider a slightly higher target range of 93–98 %. During supplementation with oxygen, it is prudent to avoid high SpO_2_ ≥ 99 % to avoid oxygen toxicity. Modified from reference [2,97].

Hyperoxemia in the early weeks of postnatal period in the very low birth weight (VLBW) infants has been known to be a major risk factor for the development of ROP [61,62]. ROP is a biphasic disorder of the retinal blood vessels that occurs at roughly two distinct time-periods (Fig. 4). In the first “vaso-obliterative” phase (between birth and ~ 30 to 32 weeks PMA), hyperoxia leads to vasoconstriction and obliteration of the developing capillary endothelial framework. In the second “vasoproliferative” phase (after ~32 to 34 weeks PMA), due to increasing metabolic demand of the developing retina in the setting of compromised vascular supply, relative hypoxia ensues leading to abnormal neovascularization that extends into the vitreous [51,63]. Based on the timing and pathogenesis of these 2 phases of ROP, several small clinical studies investigated the effects of different oxygen saturation ranges in both early and late stages of the neonatal course. In a comparative study that looked at before and after policy change of lowering the oxygen saturation alarm limit from 87 to 97 % to 85 to 93 % in infants ≤1250 g and/or ≤ 28 weeks’ gestation at birth till 32 weeks’ gestation, showed a 68 % reduction in prethreshold ROP [higher target (44/251; 17.5 % vs. lower (4/72; 5.6 %); *P* = 0.01] A study from University of Iowa evaluated the progression of ROP before and after institution of an oxygen therapy protocol. This protocol included sequential increase in SpO_2_ targets from 84 to 93 % at ≤26 weeks PMA, 86–94 % at 27–31 weeks and 90–95 % at ≥32 weeks PMA. If prethreshold or threshold ROP was diagnosed, SpO_2_ target was increased to ≥97 % (not weaning below an effective inspired oxygen concentration of 35–40 %) until ROP regression occurred. Adoption of this protocol resulted in a significant reduction in the progression from Stage 2 to Stage 3 ROP (OR 0.37, [95 % CI 0.20–0.67; *P* = 0.0013. There was no statistically significant difference between the two groups with supplemental oxygen at 36 weeks PMA, oxygen requirement at discharge or discharge pulmonary medications [64]. In the previously mentioned STOP-ROP study, a subgroup analysis showed that infants with prethreshold ROP without plus disease who were randomized to the higher oxygen saturation arm (96 % to 99 %) had significantly lower progression to threshold ROP when compared to the conventional arm (89 % to 94 %). In the HOPE-ROP (Higher Oxygen Percentage in Retinopathy of Prematurity Study) observational study (*N* = 136), infants that were excluded from the STOP-ROP (*N* = 229) trial because their median oxygen saturation by pulse oximetry (SpO_2_) values were > 94 % in room air at the time of prethreshold diagnosis, showed that the progression from prethreshold to threshold ROP trended less often than the infants in STOP-ROP who had a median SpO_2_ values ≤94 % (OR 0.607; [95 % CI 0.359–1.026)]. Subgroup analysis also showed that to be especially true for infants without plus disease [65]. A recent meta-analysis included 10 studies from 1985 to 2005 aiming to evaluate the association between the incidence of severe ROP at each of the 2 phases of the disease in premature infants (≤32 weeks’ gestation) with high or low target oxygen saturation measured by pulse oximetry and determine the optimal oxygenation range within those 2 phases. It concluded that there was a significant risk reduction of severe ROP by 52 % (RR 0.48 [95 % CI:0.31–0.75]) in the low oxygen saturation (70 % to 96 %) range in the first postnatal weeks and by 46 % (RR 0.54 [95 % CI: 0.35–0.82]) in the high oxygen saturation (>94 % to 99 %) range at PMA of ≥32 weeks. In the late high oxygen group (after PMA of 32 weeks), stratified analysis further demonstrated a stronger protective effect when the SpO_2_ lower limits were ≥ 98 % than those with 89 % to 96 % [66]. We recommend SpO_2_ target of 90–94 % in the first postnatal weeks followed by an increase to 94–98 % after 32 weeks PMA especially with the diagnosis of advanced ROP or BPD with pulmonary hypertension (Fig. 4). Such biphasic approach requires careful evaluation in a large multicenter trial.

## 8. ROP and resource limited countries: growing epidemic

**Fig. 5.**
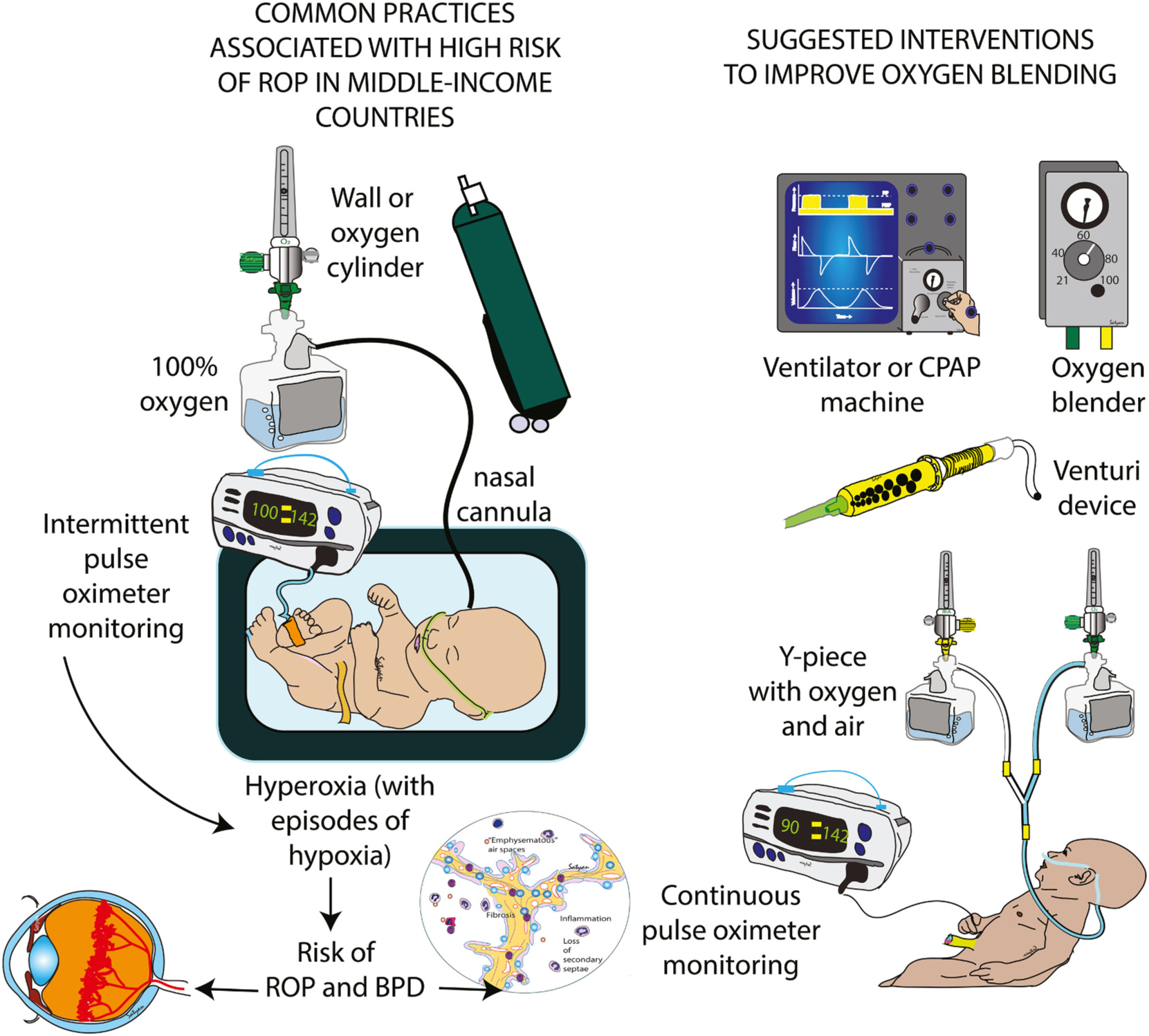
Common NICU practices associated with high risk of ROP in LMIC and suggested interventions. Use of unblended 100 % oxygen, lack of continuous pulse oximetry monitoring and episodes of hyperoxia alternating with hypoxia increase the risk of ROP. Use of oxygen blenders (either commercially available blenders or blenders linked to ventilator/CPAP machine, low-cost Venturi devices, Y-piece to mix oxygen and air with continuous pulse oximetry can minimize hypoxia and hyperoxia and potentially improve outcomes in neonates.

What the United States experienced in the early 1940s to the1950s with regards to severe ROP among survival in the preterm infants, is now being played out in other parts of the world. In countries where resources are limited, preterm births are high and expanding NICUs are built with limited integration of ROP screening and treatment protocols, the rate of ROP is increasing among survivors [67]. India, with the largest population in the world of >1.45 billion, also has the largest number of preterm births (based on modelled national estimates), accounting for almost a quarter (23.7 %) of the 14.8 million preterm births globally [68]. In 2014 it was estimated that approximately 3.5 million of the 26 million births were premature (gestational age < 37 weeks), of which an estimated 490,000 infants were born with a gestational age of <32 weeks and at least 5000 preterm infants required treatment for ROP every year [69]. To combat this problem, the Indian government supported ROP screening through the National Programme for Control of Blindness and Visual impairment launched a program to reduce blindness [70] with contribution from nongovernment eye hospitals and top government tertiary medical centers with a NICU [71]. Unlike Sub-Saharan Africa, corneal blindness is no longer a common cause of preventable childhood blindness in India. [72–74]. Lack of oxygen blenders in the NICU may lead to more ROP. With improved access to oxygen following the COVID-19 pandemic, and proliferation of NICUs, the incidence of ROP is rapidly increasing. [75,76]. Lack of oxygen blenders and inability to titrate inspired oxygen is common in resource-limited settings (Fig. 5 and supplementary table 1) due to high cost and lack of resources [99]. A brief survey of NICUs in southern Karnataka state in India by the authors (RM and KMS) showed that only 35 % of delivery rooms associated with small NICUs and 50 % of delivery rooms associated with larger level III NICUs had oxygen blenders in the delivery room. In the NICU, 27–30 % of hospitals did not use a blender while providing oxygen through a nasal cannula, mask or hood (supplementary table 1) mainly due to high cost and lack of resources. Development of low-cost blenders and innovative ways to titrate inspired oxygen based on SpO_2_ is critical to reduce the burden of ROP in LMIC (Fig. 5). Some states in India have developed innovative programs such as KIDROP in Karnataka state. KIDROP was India’s first tele-ROP program and is one of the largest single-center ROP programs in the world covering 127 NICUs in this state. [77] Such telemedicine programs that used trained personnel to screen preterm infants under supervision from pediatric ophthalmologists are critical to screen and provide early intervention to ROP in LMIC.

In 1999, the World Health Organization and the International Agency for Prevention of Blindness (IAPB) launched a global initiative called VISION 2020: The Right to Sight with the aim of eliminating avoidable blindness by the year 2020. The key objectives were summarized in the acronym ISEE: **I**ntegrate into existing health care systems, **S**ustainable in terms of money and other resources, **E**quitable care and services to all and **E**xcellence of standard of care. Such initiatives provide the groundwork for ongoing global efforts to address the remaining gaps and to develop new strategies in the fight for global eye care including ROP [78].

## 9. Optimal oxygenation of term and late preterm infants with PPHN and congenital diaphragmatic hernia (CDH)

In contrast to preterm infants, there are no clinical trials evaluating the optimal oxygenation in the management of infants >34 weeks gestation with PPHN. Guidelines from the European Pediatric Pulmonary Vascular Disease Network (EPPVDN) recommend preductal SpO_2_ of 91–95 % in PPHN. [79] Canadian guidelines for management of CDH recommend a preductal SpO_2_ range between 85 and 95 %.[80] We recommend a wider range of 90–97 % based on studies in animal models of PPHN (induced by ductal ligation and meconium aspiration) [23,81,82]or a preductal PaO_2_ between 50 and 80 mmHg (6.7 to 10.7 kPa) [2].

There are several other factors that influence optimal SpO_2_ target in PPHN. Presence of acidosis (which aggravates hypoxic pulmonary vasoconstriction) [83], therapeutic hypothermia (due to shift in oxygen dissociation curve requiring higher SaO_2_ to achieve a PaO_2_ of 50–80 mmHg or 6.7 to 10.7 kPa) [84] and possibly dark skin pigmentation (due to increased incidence of occult hypoxemia, Fig. 2) [85,86] may necessitate slightly higher SpO_2_ targets (possibly 93–98 %) to achieve optimal PaO_2_ (Fig. 4).

Pulmonary vascular resistance is influenced by alveolar oxygen (PAO_2_) and pulmonary arterial oxygen tension. [87] Hence, FiO_2_ also plays an important role in decreasing PVR. In the presence of echocardiographic evidence of PPHN, the benefits of increasing FiO_2_ to reduce PVR must be weighed against pulmonary toxicity from oxygen. In a patient with CDH with PPHN already on 80 % oxygen but with no lactic acidosis, it may be better to tolerate preductal SpO_2_ of 90 % rather than increase FiO_2_ higher due to risk of pulmonary oxygen toxicity. In contrast, an infant with severe PPHN on echocardiogram due to meconium aspiration syndrome (MAS) and HIE on therapeutic hypothermia on 30 % oxygen with preductal SpO_2_ of 90 % may benefit from an increase in inspired oxygen to 40–50 % to achieve SpO_2_ of 93–98 %, as the risk of high PVR outweighs pulmonary oxygen toxicity. [2] Hence, an individualized approach to SpO_2_ target based on FiO_2_, pH, temperature and possibly skin pigmentation based on physiology is recommended instead of a fixed target irrespective of pathophysiology. [2].

## 10. Considerations related Congenital Heart Disease (CHD)

When caring for a neonate with congenital heart disease (CHD), oxygen saturation targets should be set such that pulmonary blood flow (Qp) and systemic blood flow (Qs) are well balanced, or as close to a Qp: QS ratio of 1:1 as possible. [88] If the infant does not have intracardiac shunting present, then standard oxygen saturations (90–97 %) can be targeted as Qp:Qs will be 1:1 due to the absence of shunting. However, in “mixing” lesions, where shunting is present, providing too much supplemental oxygen can result in increased Qp. High Qp can then result in pulmonary edema or even result in decreased Qs and subsequent shock.

**Fig. 6.**
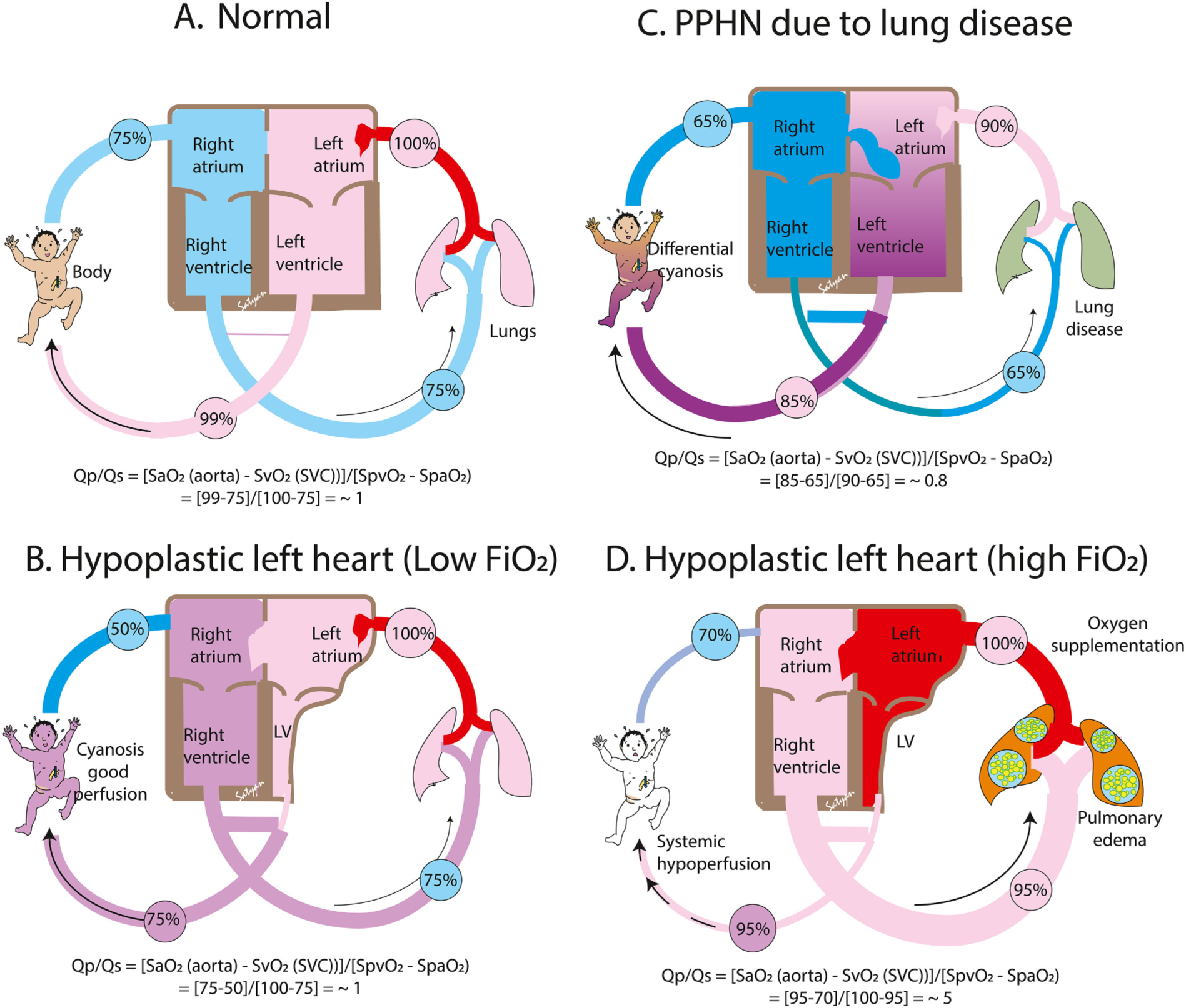
Pulmonary to systemic blood flow ratio (Qp:Qs). Calculation of Qp/Qs ratio in normal (A), an infant with severe pulmonary hypertension due to parenchymal lung disease (B), an infant with hypoplastic left heart (HLH) on low inspired oxygen (C) and high oxygen (D) are shown. SaO_2_ – arterial oxygen saturation by co-oximetry, SVC – superior vena cava, SpvO_2_ – oxygen saturation in pulmonary veins; SpaO_2_ (oxygen saturation in the pulmonary artery), LV – left ventricle.

One of the most complex mixing heart defects in the neonatal period is Hypoplastic Left Heart Syndrome (HLHS) or other single ventricle defects. When caring for a neonate preoperatively or following stage I palliation, SpO_2_ target should be 75–85 % to help balance Qp:Qs. Using Fick principles of oxygen consumption, we can arrive to an equation for Qp/Qs = (Oxygen saturation Aorta – Oxygen saturation SVC)/(Oxygen saturation Pulmonary Vein – Saturation Pulmonary Artery, Fig. 6). In an infant with fully mixing single ventricle anatomy, saturation of the aorta is equal to that of the pulmonary artery. Thus, assuming approximately 25 % oxygen consumption resulting in SVC saturation of 50 % and assuming 100 % pulmonary vein saturation, targeting SpO_2_ of 75 % results in 1:1 Qp:Qs((0.75–0.5)/(1–0.75)=1) as shown in Fig. 6B. Whereas, increasing SpO_2_ to 95 % would increase mixed the venous saturation, would increase Qp:Qsto5((0.95–0.7)/(1–0.95)=5), and result in pulmonary edema and may even lead to shock from compromised Qs as shown in Fig. 6D. Thus, avoiding unnecessary supplemental oxygen is crucial in these patients. Approaches to augment Qs, such as administration of milrinone to lower systemic vascular resistance and possibly promote more Qs may be helpful. However, medical interventions to reduce Qp when a preoperative neonate is saturating over 85 %, such as blending in other gases to lower FiO_2_, have not been widely studied or used clinically.

## 11. Monitoring SpO_2_ trends with a histogram

The use of a continuous pulse oximetry as a non-invasive method of measuring oxygen saturation is now almost universal in the NICU. It allows clinicians to continuously monitor levels of oxygen saturation and therefore be able to target a defined goal range. However, without frequent surveillance and analysis of the data this may in fact diminish any benefits of targeted oxygenation parameters. The success of maintaining the intended pulse oximetry saturation range can vary substantially among different NICUs with one study showing infants <28 weeks’ gestation in the first 4 postnatal weeks SpO_2_ was outside the range of intention more than half of the time despite having unit policy in place [89]. With such variation in achieved versus intended results, practices that utilize close-loop servocontrol systems that pair SpO_2_ feedback to the delivery of FiO_2_ showed more effectiveness than routine manual oxygen manipulation in maintaining target oxygenation saturation levels [90–92]. Achieving goal oxygen saturations is a constant challenge for the bedside provider but quality improvement (QI) initiatives that places an emphasis on histogram analysis with multiple Plan-Do-Study-Act (PDSA) cycles to identify interventions to improve achieved oxygen saturations in preterm infants, has been associated with a reduction in death or severe ROP [93].

## 12. Conclusion

Oxygen is one of the most commonly used “drugs” in the NICU. It has a narrow therapeutic window. Titration of inspired oxygen and maintenance of SpO_2_ within a specified range based on infant’s gestational age, postnatal age and pathophysiology is critical to avoid hypoxia and minimize oxygen toxicity. The advent of automated inspired oxygen controls may enhance our ability to maintain target oxygenation. [94] Creation of low-cost devices to blend and monitor oxygenation are needed to limit hypoxia and the effects of oxygen toxicity in LMICs.

## Supplementary Material

Supplement Table 1
