# Supplementary material for: Oxygen saturation targets in neonatal care: A narrative review
Source: Early Hum Dev. Author manuscript; Available in PMC 2025 Dec 1. (PMC12121305; doi:10.1016/j.earlhumdev.2024.106134)
Supplement: Supplement Table 1 [file NIHMS2081265-supplement-Supplement_Table_1.docx]

Table 1. Oxygen administration and monitoring among level 1-3 NICUs from the state of Karnataka, India; Data shown as median (interquartile range) or n (%)

| NICU admissions | < 50 per month (n=23) | ≥ 50 per month (n=22) |
| --- | --- | --- |
| NICU level   - Level 1 - Level 2 - Level 3 | 2 (9%)  4 (17%)  17 (74%) | 22 (100%) |
| Number of NICU beds | 5 (3-8) | 25 (10-42) |
| Number of NICU admissions per month | 15 (10-30) | 100 (68-200) |
| Inborn NICU admissions | 10 (10-30) | 81 (45-146) |
| NICU admissions < 1500g | 3 (2-7) | 30 (20-65) |
| Number of babies requiring resuscitation per month | 5 (3 – 8) | 25 (10- 42) |
| Oxygen blender in DR | 35% | 50% |
| Type of oxygen blender used in DR   - Commercially available blender - Manual Y-piece - Others | 89%  0%  11% | 82%  9%  9% |
| Reason for not having a blender: lack of resources | 73% | 64% |
| Pulse oximeter monitoring in the DR | 82.6% | 86.3% |
| NICU oxygen use:   - oxygen blender for nasal cannula, mask or hood - No blender and 100% oxygen use for these devices | 69.6%  30.4% | 63.6%  27.4% |
| Type of oxygen blender used in the NICU   - Commercially available blender - Blender supplied with CPAP/ventilator - Low-cost blender - Manual Y-piece | 29.4%  58.8%  5.9%  5.9% | 31.2%  68.8% |
| Number of ROP cases per year | 2 (0-7) | 30 (6-165) |
